# Editorial: The brain meets the body: neural basis of cognitive contribution in movement for healthy and neurological populations

**DOI:** 10.3389/fnhum.2023.1306252

**Published:** 2023-10-18

**Authors:** Valeria Belluscio, Viviana Betti, Alex Martino Cinnera, Daniela De Bartolo

**Affiliations:** ^1^Department of Movement, Human and Health Sciences, Interuniversity Centre of Bioengineering of the Human Neuromusculoskeletal System, University of Rome “Foro Italico”, Rome, Italy; ^2^IRCCS Fondazione Santa Lucia, Rome, Italy; ^3^Department of Psychology, Sapienza University of Rome, Rome, Italy; ^4^Department of Human Movement Sciences, Faculty of Behavioural and Movement Sciences, Amsterdam Movement Sciences and Institute for Brain and Behaviour Amsterdam, Vrije Universiteit Amsterdam, Amsterdam, Netherlands

**Keywords:** brain dynamics, motor control, measurement, cognitive-motor interplay, neuroscience

## Cortical activation in movement

The human brain comprises billions of neurons forming an intricate network of interactions (Baars and Gage, 2010). Our understanding of brain function has progressed in recent decades (Sakkalis, 2011), delving into the basis of human behavior and function.

Most of everyday human movements are deceptively complex and only appear easy because of extensive practice. Cortical and subcortical networks, known to be involved in the planning and execution of movement (Meirhaeghe et al., 2023), are difficult to assess with imaging techniques in real-time (Sisti et al.). Therefore, modern research is increasingly interested in understanding this mechanism through dual-task conditions, exploiting external stimulations, such as acoustic or visual stimuli, to investigate cortical functioning in motor tasks execution (Bajaj et al., 2015; De Bartolo et al., 2020, 2021; Verna et al., 2020). In this Research Topic, Li et al. investigated whether Tai Chi (TC) practice can improve the brain connectivity of the prefrontal lobe. The authors showed a positive effect of TC on the frontal and temporal networks associated with decision making and abstract thinking. Furthermore, this study investigated the role of cognitive control in the realization of a motor performance, thus linking the improved activation of the prefrontal lobes to the enhancement of the brain functional neuroplasticity. Therefore, authors suggested that TC has a crucial role in improving the physical and mental health of college students, providing scientific guidance for the promotion of TC on campus.

Using fNIRS and virtual reality (VR), Zheng et al. detected the activation of the cerebral cortex under different VR interaction modes, exploring the optimal feedback associated to the improvement and the effectiveness of rehabilitation training with robots. Authors evaluated the effects of multiple VR interaction modalities based on force-haptic feedback combined with visual or auditory stimuli, finding that the multisensory integration is conducive to a stronger cortical activation due to the interaction effect between visual and auditory feedback. These results provided a theoretical basis for the optimal design of rehabilitation robots thus formulating potential VR-based clinical schemes.

Song et al. detected with fNIRS a strong network activity in the bilateral prefrontal, motor, and occipital cortex following a motor exertion based on a race-walking task. These real-time changes reflected different brain network-specific characteristics, suggesting that a more extensive brain activation is needed to process information about speed. Authors suggested that increased motor activity may facilitate the integration of proprioception and motor planning involved in coordinated actions. Finally, in this Research Topic Strong et al. used fMRI to identify brain regions associated with the proprioceptive sense of joint position of the knee in people with injury to the anterior cruciate ligament. Using a knee joint position sense test during simultaneous fMRI, the authors observed significant correlations with increased ipsilateral response in the anterior cingulate, supramarginal gyrus and insula, and sensorimotor processes, body schema and interoception These results provided further insights into brain response to proprioception among different populations.

## Postural control and the neuro-muscular investigation

Regulation and control of posture represent a challenge for the body's neuromuscular control system (Hayes, 1982; Munoz-Martel et al., 2019; Martino Cinnera et al., 2022) and its dysfunction affects the daily living activity of neurological patients (Nonnekes et al., 2018). The review proposed by Chaudhary et al., addressed the role of the visual system and its interaction with the vestibular system to maintain postural stability. Authors outlined how visual-vestibular interactions enhance postural stability by interpreting the head's position and generating eye movements accordingly, which helps differentiate self-motion or external motion and achieve gaze stabilization and postural control. Indirect measurement of brain responses to postural changes is also addressed through the assessment of neuromuscular and motor functions, as in the investigation of defense mechanisms adopted to keep the body away from the stimulus (Graziano and Cooke, 2006; Belluscio et al., 2023). In humans the space directly surrounding the body at a grasping distance has been depicted as defensive peripersonal space for tactile (Rizzolatti et al., 1997) or auditory stimulation (Ladavas et al., 2001), closer the stimulation to the head stronger behavioral response was found (Versace et al., 2019). Hamada et al. used EMG to investigate how the prediction of an auditory stimulus site of the head may induce a defense response of the body swaying in the opposite direction, unraveling the specific contribution of the gastrocnemius muscle.

Zipser-Mohammadzada et al. investigated the relationship between intramuscular coherence and corticospinal dynamic balance control during a visually guided walking treadmill task through EMG recordings in a group of incomplete spinal cord injury patients (iSCI). This study provided a reliable measure of adaptive walking ability in individuals with iSCI, thus contributing to new knowledge about supra-spinal locomotor control.

Finally, Schueren et al. explored the role of a somatosensory illusion influencing the postural after-effects of standing on an inclined surface. Using a force plate, the authors showed that lower extremity manipulation is a useful intervention to treat postural stability. This study contributes to the growing body of evidence that manipulation of the lower extremities can drive global postural changes, as well as influence standing behavior.

This Research Topic collected significant contributions to the advancement of the motor-cognitive interplay. The integrative use of novel neuroscientific techniques strives for an in-depth investigation of motor behavior, assuming a multidisciplinary approach to the study of brain–behavior relations also in neurological populations. This will enable neuroscientific knowledge to be effectively translated for clinical practice.

## Author contributions

VBel: Methodology, Writing—original draft, Writing—review and editing. VBet: Writing—review and editing. AM: Writing—review and editing. DD: Writing—review and editing, Conceptualization, Investigation, Methodology, Project administration, Supervision, Writing—original draft.
